# Development and preliminary evaluation of a ΔtssB-EP as a live attenuated vaccine against Edwardsiella piscicida

**DOI:** 10.3389/fimmu.2026.1813826

**Published:** 2026-08-13

**Authors:** Rakesh Das, Cheng Xu, Koushik Ghosh, Noora Barzkar, Abhisek Mukhopadhyay, Øystein Evensen

**Affiliations:** 1 Norwegian University of Life Sciences, Faculty of Veterinary Medicine, Ås, Norway; 2 ICAR-Central Institute of Freshwater Aquaculture, Odisha, India; 3 Aquaculture Laboratory, Department of Zoology, The University of Burdwan, Burdwan, West Bengal, India; 4 Higher Institution Centers of Excellence, Borneo Marine Research Institute, Universiti Malaysia Sabah, Sabah, Malaysia

**Keywords:** aquaculture, bacterial diseases, Edwardsiellosis, in-frame deletion, preventive measures, vaccine

## Abstract

**Background:**

Vaccination is the most effective strategy for preventing and controlling infectious diseases in aquaculture. *Edwardsiella piscicida*, a Gram-negative facultative intercellular bacterial pathogen, is the etiological agent of edwardsiellosis in fish, a disease associated with significant morbidity, mortality, and economic losses in the aquaculture industry.

**Methods:**

In this study, a live attenuated mutant strain, designated ΔtssB-EP, was generated from a virulent Edwardsiella piscicida by in-frame deletion of the tssB gene, which encodes a component of the Type VI secretory system, using an allelic exchange approach. A series of *in vitro* and *in vivo* studies was conducted to evaluate the potential of the developed mutant strain as an effective vaccine candidate.

**Results:**

The precise deletion of about 506 bp was confirmed by PCR. The studies demonstrated that the ΔtssB-EP (mutant) exhibited a significant reduction in biofilm formation, along with a 2.7 log_10_ increase in lethal dose (LD_50_) compared to the wild-type strain. In addition, a vaccination trial was conducted in which *Labio rohita* juveniles were immunized via intraperitoneal (I.P.) injection with a sublethal dose of the ΔtssB-EP mutant. At 35 days post-vaccination (dpv), the fish were challenged with wild-type *E. piscicida* to evaluate protection against mortality. After vaccination, antibody levels were significantly higher in the vaccinated group than in the control group (PBS/Mock-vaccinated). The improved relative percentage survival (RPS = 70.83%) was observed in the group of *L. rohita* juveniles administered with the ΔtssB-EP mutant.

**Conclusion:**

The results confirm significant attenuation of virulence in the ΔtssB-EP mutant, which elicited a robust immune response and conferred substantial protection against mortality from *E. piscicida* infection in Labeo rohita.

## Highlights

- ΔtssB-EP was constructed on the basis of an allele-exchange protocol, and the precise deletion of about 506bp in the tssB gene of Edwardsiella piscicida was confirmed by PCR.
- ΔtssB-EP exhibited a significant reduction in biofilm formation, along with a 2.7 log_10_ increase in LD_50_ compared to the wild-type strain.
- After vaccination, significantly higher IgM levels were observed in the vaccinated group compared to the control group.
- A relative percentage survival, RPS, of 70.83% was observed in the group of *L. rohita* juveniles immunized with the ΔtssB-EP (mutant strain).

## Introduction

Infectious diseases represent a significant challenge to the aquaculture industry, causing substantial economic losses due to increased morbidity and mortality (1). Vaccination is a widely adopted disease management strategy in aquaculture globally, playing a key role in reducing antibiotic use in fish farming. The successful application of vaccination strategies in aquaculture depends on three critical factors: cost-effectiveness, safety, and practicality. Therefore, comprehensive screening procedures are essential to identify live attenuated vaccines that are both effective and stable, and suitable for application in aquaculture systems (2). An ideal fish vaccine should be safe for both the target animal and the environment, cost-effective for large-scale production, simple to administer, capable of eliciting robust and long-lasting immunity during periods of highest susceptibility, and associated with minimal adverse effects (3). Furthermore, improving the sustainability of finfish production requires addressing critical issues affecting fish welfare, economic stability, and ecological balance. These challenges drive the development of novel vaccines and the implementation of effective immunization strategies targeting both emerging and persistent diseases in aquaculture (4).

*E. piscicida* is a gram-negative, facultatively anaerobic bacterium that causes Edwardsiellosis in several commercially important fish species and imposes significant financial losses in aquaculture systems (5). According to Shafiei et al. (6), edwardsiellosis has spread to multiple countries across Asia, Europe, and the United States. To date, species such as catfish (*Ictalurus furcatus*), tilapia (*Oreochromis niloticus*), turbot (*Scophthalmus maximus*), and other freshwater and marine fish species important to the aquaculture industry have been affected by Edwardsiellosis (7). Diseased fish exhibit a range of clinical manifestations, including diminished pigmentation, exophthalmia, ocular opacity, cutaneous lesions, petechial bleeding, and pathological changes such as liquefaction and necrosis in vital organs, including the kidney, spleen, and liver (8). Antibiotic use in aquaculture exerts selective pressure that promotes the emergence of drug-resistant bacterial reservoirs and the dissemination of transferable resistance genes in the aquatic environment (9). Hence, there is an urgent need to develop alternative control measures, such as rationally designed vaccines, to combat emerging bacterial pathogens.

The Type VI Secretion System (T6SS) is a critical virulence-related secretion mechanism in Edwardsiella spp., facilitating bacterial adherence, intracellular persistence, multiplication within phagocytic cells, host colonization, immunological regulation, and interbacterial competition. Prior research has shown that disruption of T6SS-related genes, particularly those within the Evp gene cluster, markedly diminishes bacterial virulence and impedes host-pathogen interactions in both piscine and mammalian models (8, 10). T6SS effector proteins, including EvpP, are crucial for macrophage survival, intracellular replication, and the control of host immunological responses (11). TssB (EvpB), a fundamental structural protein, is a component of the contractile sheath complex, which is essential for the proper assembly and functional secretion of the T6SS, making it a promising target for attenuation and live vaccine development. In addition, T6SS is a critical virulence determinant in numerous pathogenic Gram-negative bacteria (12). It is highly conserved and widely distributed amongst diverse Gram-negative bacteria, typically present in one or multiple copies within a genome (13). T6SS mediates the direct delivery of protein effectors into the periplasm of target cells, contributing to both interbacterial competition and host pathogenicity. T6SS gene clusters typically comprise 16 to 38 genes (14), with at least 13 core components required for the assembly of a functional secretion apparatus (15, 16). T6SS plays a pivotal role in bacterial antagonism by delivering antibacterial effectors that promote the elimination of competing bacterial cells (17).

*Labeo rohita* (rohu), one of the principal Indian major carps (IMCs), is a highly favored species in carp polyculture due to its rapid growth, adaptability, and it has been spread across South and Southeast Asia, including Sri Lanka, Nepal, China, Japan, the Philippines, and Malaysia, as well as in regions of the former USSR and several African nations (18). However, disease outbreaks in carp polyculture farms, particularly in India, have been associated with suboptimal management practices, leading to the rapid proliferation of bacterial pathogens that exploit these conditions (19). In this study, *L. rohita* was used as a model species to evaluate the efficacy of a rationally designed live attenuated vaccine under controlled wet-laboratory conditions. The vaccine candidate, a mutant strain of *E. piscicida* ΔtssB-EP, was generated via homologous recombination. Its safety and elicited protection against lethal challenge were assessed through intraperitoneal immunization followed by a challenge trial using a virulent wild-type *E. piscicida* strain.

## Materials and methods

### Animal ethics

All experiments were completed in accordance with the guidelines provided in ARRIVE 2.0 20 (Percie de Sert). Every procedure was performed under humane conditions to minimize any potential distress to the experimental animals. The experimental protocol involving *L. rohita* juveniles was approved by the Institutional Ethics Committees, i.e., the Institutional Biosafety Committee (BU/IBSC/19/13(33) dt. 23-05-2019) and the Dissection Monitoring Committee (BU-DMC/2021/01/06 dt. 07-10-2021) of The University of Burdwan, West Bengal, India. In addition, all fish-related experiments were conducted in accordance with EU directives on the humane killing or sacrifice of animals, without causing any stress. Further, sufficient care was taken, and no stress was imposed during the wet laboratory/immunization trials.

### Fish

Healthy *Labeo rohita* (average weight 11 ± 0.5 g) juveniles were collected from a commercial fish farm without any previous disease history or clinical signs/symptoms. Upon arrival, the fish stocks were kept in an indoor facility with running aerated water at 28 ± 2 °C and fed commercial feed at 2% of body weight twice daily for about two weeks. Fish were screened for infections or etiological agents on alternate days during the acclimatization period at the wet lab facility.

For the collection of tissue samples, the fish were first anesthetized with 100 mg/L of anesthesia, prepared as a 1:1 w/w mixture of 5.0 g ethyl-3-aminobenzoate methane sulfonate (Sigma-Aldrich, Cat. No. E10521) and 5.0 g sodium bicarbonate (Sigma-Aldrich, Cat. No. S5761) in a total of 50L of water and left for a minimum of 5 minutes in the solution. Once the fish reached deep anesthesia, they were euthanized with a sharp blow to the head, followed by dissection and sample collection.

### Bacterial strains, plasmids, and growth conditions

*Edwardsiella piscicida* (strain WSFC2; NCBI accession No. JAPMJQ000000000) was previously isolated from infected fish and stored in glycerol stock at -80 °C at the Norwegian University of Life Sciences. For revival, it was aseptically cultured at 28 °C in tryptic soy agar (TSA) or tryptic soy broth (TSB). The Escherichia coli S17-1 λpir, used for plasmid mobilization, was grown at 37 °C in LB broth or on LB agar. Ampicillin (100 µg/mL) (Sigma-Aldrich) and chloramphenicol (20 µg/mL) (Sigma-Aldrich) were used for selection.

### Construction of the ΔtssB-EP mutant strain

**Figure 1:**
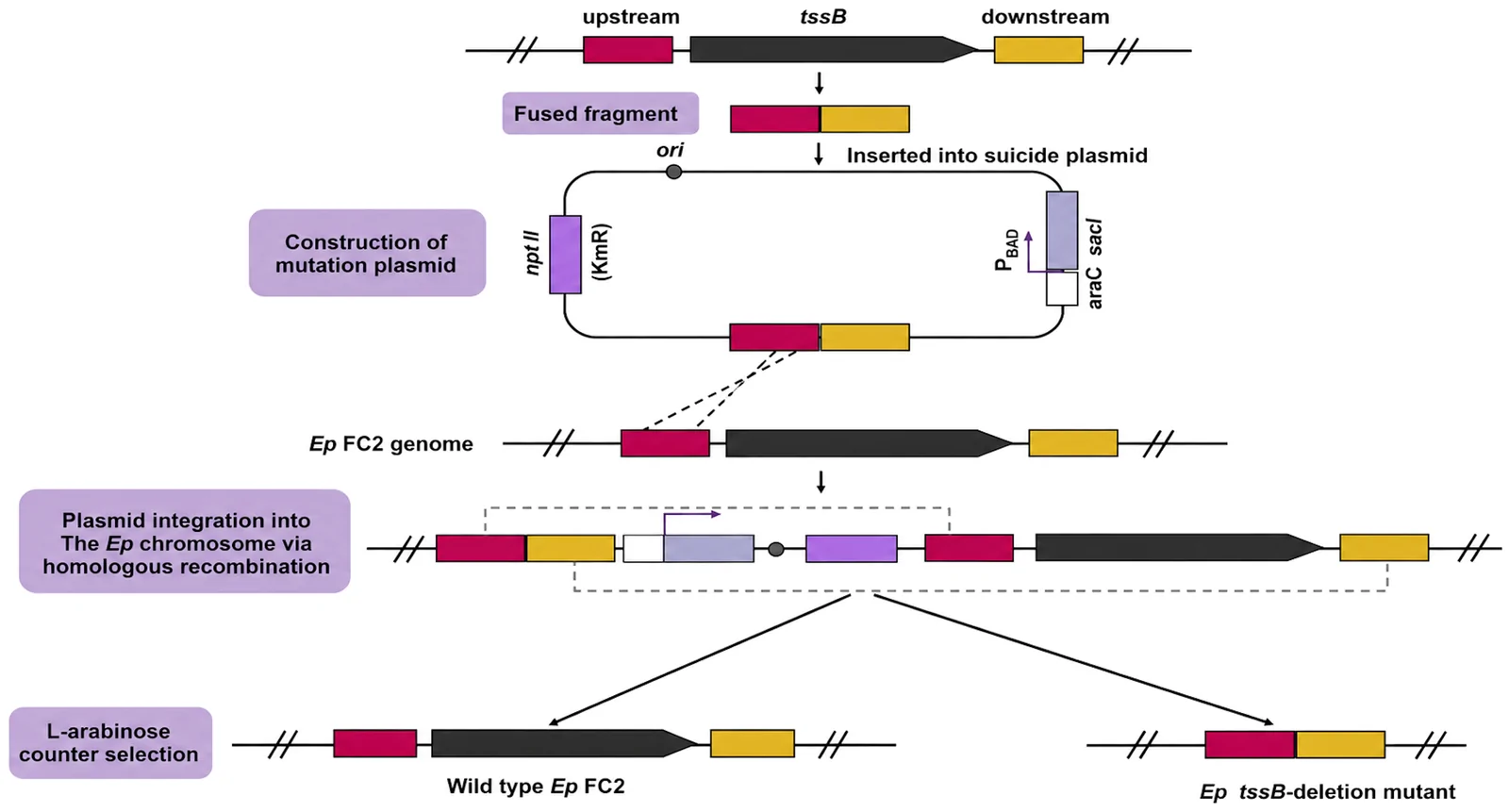
Schematic representation of the two-step allelic exchange protocol followed in the current study.

An in-frame deletion mutant of *E. piscicida* targeting the tssB gene (designated as ΔtssB-EP) was constructed using a two-step allelic exchange strategy (**Figure 1**). Briefly, approximately 500 bp DNA fragments corresponding to the upstream and downstream flanking regions of the tssB locus were amplified separately by PCR and subsequently fused through overlap extension PCR using Phanta^®^ Max Super-Fidelity DNA Polymerase (Vazyme Biotech, China). The fused PCR product generated an in-frame deletion cassette lacking the internal coding region of tssB while preserving the translational reading frame to minimize potential polar effects on adjacent genes. The purified deletion cassette was digested with XhoI and XbaI restriction enzymes and ligated into the suicide plasmid pTL1010, yielding the recombinant plasmid pTL1010-ΔtssB. The suicide vector pTL1010 harbors an arabinose-inducible counter-selection system based on the C-terminal DNase domain of the Type VI secretion system toxin Txe1, enabling efficient selection of double-crossover recombinants.

After that, the recombinant plasmid was mobilized into *E. piscicida* strain WSFC2 via biparental conjugation using Escherichia coli S17-1 λpir as the donor strain. Transconjugants carrying the integrated plasmid were initially selected on appropriate antibiotic-supplemented media. Subsequently, a second homologous recombination event was induced through counter-selection on L-arabinose-containing medium, facilitating excision of the plasmid backbone and generation of the markerless ΔtssB mutant. Multiple colonies surviving counter-selection were screened by colony PCR with external flanking primers (tssb-US-F and tssb-DS-R) to identify successful deletion mutants by the expected reduced amplicon size. To further verify the identity of the mutant strain as *E. piscicida*, species-specific PCR amplification targeting the etfD gene was performed with the primer pair etfD-F and etfD-R. Furthermore, three independent allelic exchange experiments were performed to assess reproducibility. Consequently, more than 20 colonies were screened each time to confirm the deletion. Furthermore, all PCR-amplified and gel-purified products were thoroughly confirmed by Sanger sequencing. The list of primers used in the study has been provided in **Table 1**.

**Table 1:** Primers details.

Primers	Sequence	Description	Amplicon size	References
tssb-US-F	AATTCTAGAAAGCGTATGCTGGCTAAGCAA (XbaI)	To amplify the upstream fragment of tssB	561 bp	This study.
tssb-US-R	TGTTCGCTCATGGCATGCATCTCCGTAACATTTCTTACAAC			
tssb-DS-F	GATGACTTCATGCCATGAACAGAACTTGCCA	To amplify the downstream fragment of tssB	547 bp	This study.
tssb-DS-R	AATCTCGAGTGTGAGATCCAGCAGGAACA (XhoI)			
etfD-F	GGTAACCTGATTTGGCGTTC	To verify E. piscicida strains	445 bp	(21)
etfD-R	GGATCACCTGGATCTTA CC			

1 (US, Upstream; DS, Downstream; F, Forward; R, Reverse).

### Biofilm formation assay

The biofilm-forming capacity of the wild-type and mutant strains of *E. piscicida* was investigated using the procedure outlined by Swain et al. (22) with minor adjustments. In brief, the overnight cultures were diluted 1:100 in LB and grown at 28 °C to an OD_600_ ≈of 0.5, then further diluted 1:20 in fresh LB. Aliquots (200 µL) were added to 96-well plates (five replicates per sample; sterile LB as negative control) and incubated statically at 28 °C for 48 h. After incubation, OD_600_ was recorded. Wells were washed twice with 1× PBS, fixed with 200 µL methanol for 15 min, air-dried at 37 °C, and stained with 0.5% crystal violet for 15 min. Excess stain was removed by rinsing with distilled water. Bound dye was solubilized with 95% ethanol for 15 min, and OD_570_ was measured using an ELISA plate reader. Biofilm formation was expressed as the OD_570_/OD_600_ ratio.

### Virulence determination and estimation of median lethal dose (LD50) of *E. piscicida* mutant (ΔtssB-EP)

**Figure 2:**
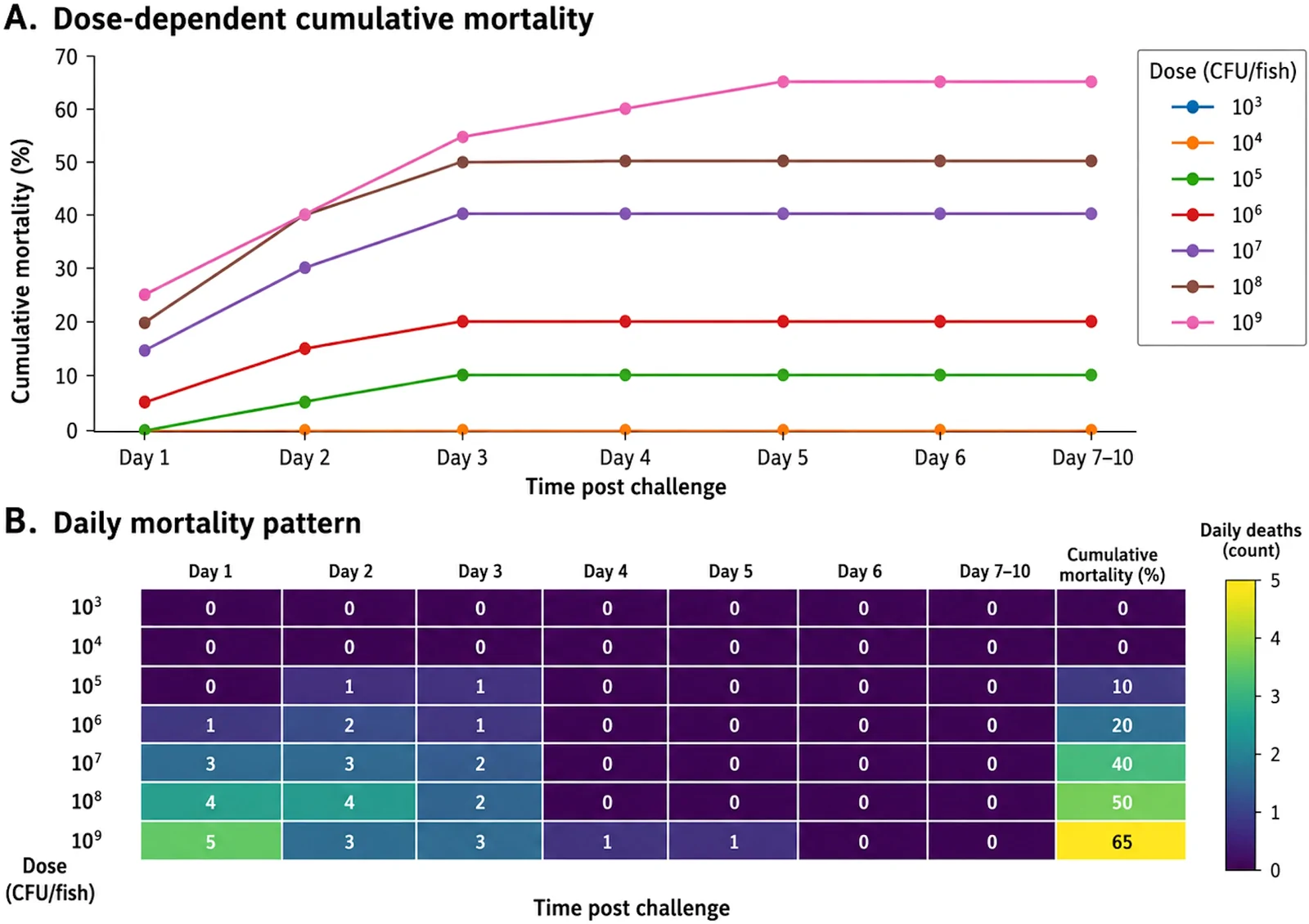
**(A)** Dose-dependent cumulative mortality (%) following challenge with the mutant strain (ΔtssB-EP) at different doses, 10^3–^10^9^ per fish. **(B)** Daily mortality from day 1–10 post-challenge, with percent cumulative mortality showing daily death counts. Each dose group contained 20 fish.

The median lethal dose (LD50) method was used to assess the virulence of ΔtssB of E. piscicida (ΔtssB-EP) relative to the wild-type (WT) strain of E. piscicida. Healthy *L. rohita* were injected intraperitoneally (I.P.) with a gradient of 10-fold serial dilutions ranging from 10^3–^10^9^ colony-forming units (CFU/fish) of mutant ΔtssB-EP strain. The control group was injected with an equivalent volume of sterilized phosphate-buffered saline (PBS, pH 7.4). For mutant and WT, fish were randomly divided into eight groups. Each group consisted of 120 fish and was kept in a separate 1000 L tank. The fish were observed for 10 days, and all dead fish were sampled to detect *E. piscicida*. Mortalities were recorded (**Figure 2B**), and the LD_50_ was calculated using Reed and Muench’s (23) method.

### Evaluation of vaccine protection

#### Immunization trial

Each group was housed in an individual tank in triplicate throughout the experiment to prevent the spread of infection between groups. For 3 weeks, fish were fed at 3% of their body weight twice daily, specifically at 9:00 and 17:00 hrs. The feed quantities were modified every two weeks to accommodate the updated fish biomass. Daily, the fish waste was removed, and half of the water was replaced with aerated, dechlorinated water from the storage tank. For the vaccination trial, the acclimatized fish were randomly distributed without any specific sex ratio in the experimental tank (1000 liters) in triplicate (n= 45 each) and intraperitoneally (i.p.) injected with 100 μL of the mutant strain (ΔtssB-EP) suspended in sterile PBS (pH 7.4) in the vaccinated group. The inoculation dose was about 1 × 10^4^ CFU/fish. The (T0) control group received a sterile PBS (1x) intraperitoneal (I.P.) injection as a negative control. The duration of immunization was prolonged to 35 days (5 weeks). The water temperature and dissolved oxygen (DO) were checked bi-daily at 8:00 and 16:00, whereas the remaining water quality indicators were measured weekly. The water temperature was maintained at 28 ± 1 °C, while the dissolved oxygen (DO) level was measured at 6 ± 0.2 mg/L. The pH value was 7.5 with a standard deviation of ±0.5. All these characteristics fulfilled the optimal conditions for promoting fish growth.

#### Total serum IgM level estimation

The total serum IgM levels were quantified using a commercial enzyme-linked immunosorbent assay (ELISA; CSBE-12045Fh, CUSABIO BIOTECH Co., Ltd., China) in accordance with the manufacturer’s instructions. Two fish samples from each tank were randomly collected and used to obtain serum samples after immunization at weekly intervals for 5 weeks (dpv).

### Challenge study

After 35 days (5 weeks) of vaccination or immunization, 20 fish from each treatment group (with triplicates), including the control group (PBS), were injected intraperitoneally (I.P.) with 0.1 ml of the virulent strain of *E. piscicida* WSFC2 (NCBI accession no. JAPMJQ000000000.1) at 1 × 10^8^ CFU/fish. An additional 20 fish were selected from the control group and used as a positive control group to assess disease signs after injection with the wild-type strain of *E. piscicida* WSFC2. For the latter, the characteristic symptoms of diseased fish included visible external bleeding, inflammation, and ulcers in the gills, fin bases, and other tissues. Deaths were recorded every 12 hours for 10 days, and the total number of deaths was calculated to estimate the cumulative mortality rate. The survival rate was determined using the relative percentage of survival (RPS) formula developed by Amend (24):

RPS is calculated as follows: $(1-\frac{mortality\;in\;vaccinates}{mortality\;in\;controls})\times 100$

### Statistical analysis

All data analysis and graph plotting were performed using GraphPad Prism software (version 10.1.2; San Diego, CA, USA). Data are expressed as the mean ± standard deviation (SD) from three separate biological replicates. Variations in IgM antibody levels (ELISA) were assessed using Two-way ANOVA, followed by Tukey’s multiple comparisons test. A p-value of less than 0.05 was deemed statistically significant. Post-challenge survival analysis was performed using the Kaplan-Meier method.

## Results

### Homologous recombination and construction of the mutant

We constructed a ΔtssB-EP mutant strain by allelic replacement as per the proven protocol 25 (Hmelo et al). with some modifications (**Figure 1**). Further, the construction of the *E. piscicida* ΔtssB-EP was done through suicide plasmid-mediated homologous recombination. The genotype was confirmed via PCR and by Sanger sequencing.

### Confirmation of partial deletion of the tssB gene

The upstream fragment corresponds to the sequence immediately upstream of the tssB coding region, and the downstream fragment corresponds to the sequence immediately downstream of tssB. These two fragments were fused by overlap PCR to generate an in-frame deletion construct in which only the tssB coding sequence is deleted following allelic exchange via homologous recombination and L-arabinose counter selection, while the neighboring genes tssA (upstream) and tssC (downstream) remain intact. We have confirmed the precise deletion of tssB and the integrity of the flanking regions by PCR and DNA sequencing; no unintended deletions or mutations were detected in tssA or tssC. We amplified DNA fragments flanking the upstream and downstream regions of tssB in the wild-type strain. The PCR product from the wild-type strain (containing the upstream and downstream regions plus tssB) was 1615 bp, whereas the PCR amplicon from the mutant strain (containing only the upstream and downstream regions) was 1109 bp. This size difference indicates that the tssB gene was knocked out in the mutant strain.

**Figure 3:**
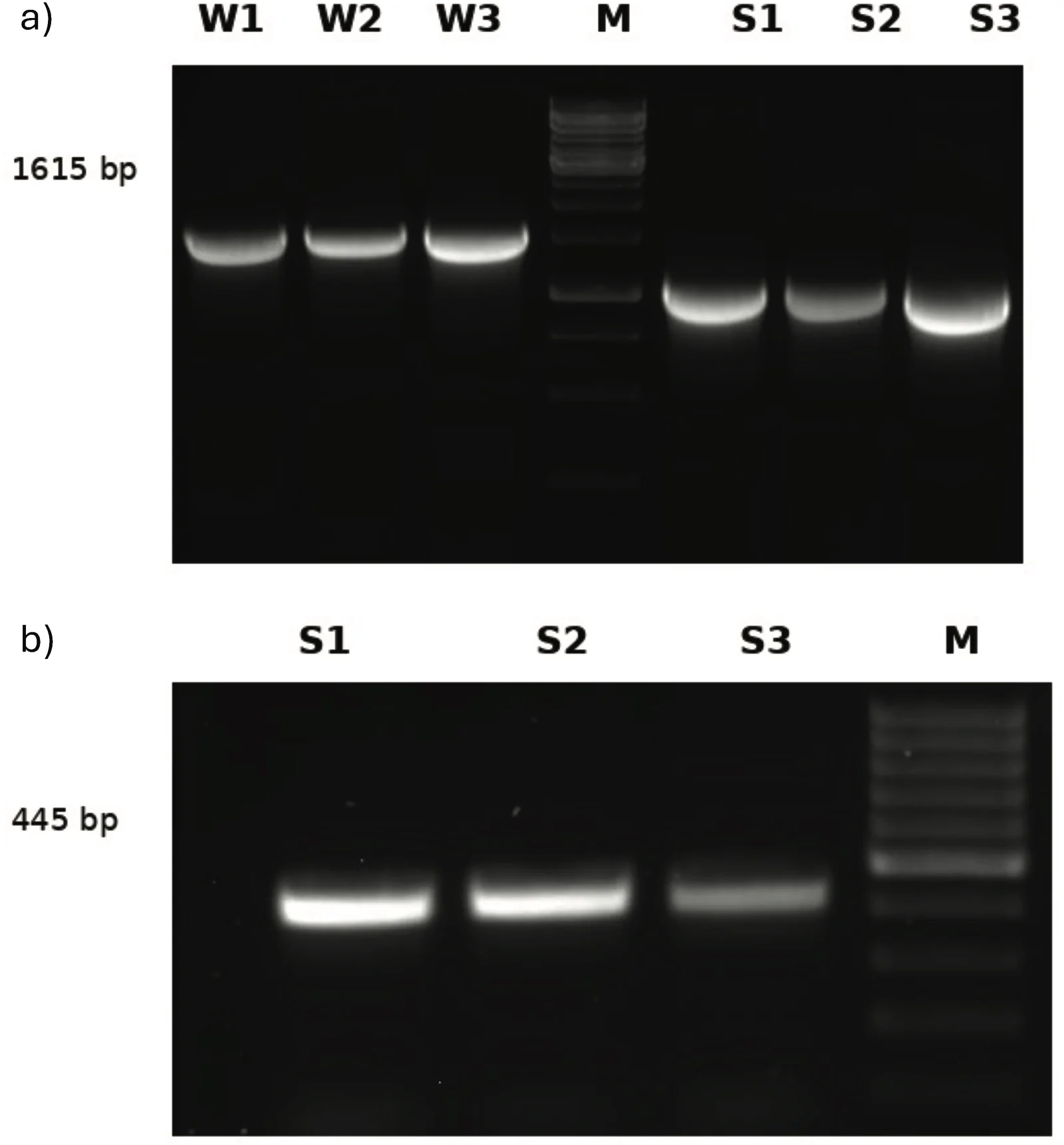
**(a)** W1-W3: The PCR product from the wild-type strain (containing the upstream and downstream regions plus tssB) is 1615 bp. S1-S3: The PCR product from the mutant strain (containing only the upstream and downstream regions) is 1109 bp. (mutant); M = 1kb ladder. **(b)** Species-level confirmation of mutant E. piscicida as a visible band at 445 bp; M = 100bp ladder.

Further, deletions in the mutant strains were verified by PCR, in which the size of the PCR products of mutant strain was smaller than that of the WT *E. piscicida* (**Figure 3a**). The differences in the bands are found at about 506 bp, which further confirms the deletion/attenuation of the wild strain in the designated gene component of the T6SS of *E. piscicida* genome (**Supplementary Material 1**). Further, to confirm the mutants derived from the *E. piscicida* strain (**Figure 3b**), species-specific primers were used to run PCR, and the expected band sizes were observed and reconfirmed after sequencing.

### Biofilm formation

**Figure 4:**
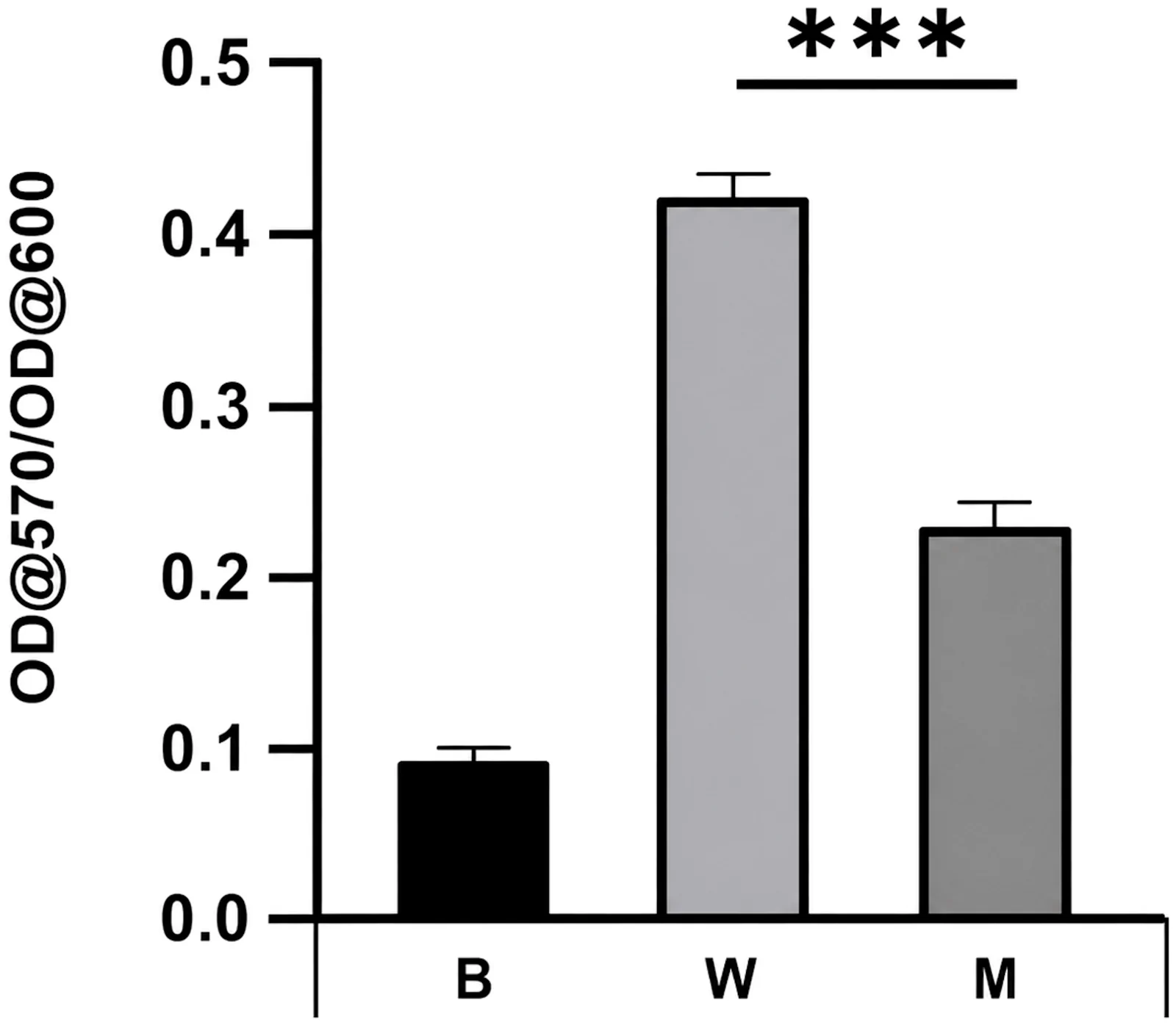
Biofilm formation ability of wild and mutant strains. Data are expressed as the mean ± SD of 3 measurements per time point. ***p< 0.001. B, Blank; W, Wild strain; M, Mutant Strain.

The ΔtssB-EP lost the capacity to form an adequate biofilm compared to the wild strain. The mutant strain, compared with the wild strain, showed a significant reduction (**Figure 4**).

### Pathogenicity of the mutant strain

The median lethal dose was determined to confirm the pathogenicity of the newly constructed mutant. The LD_50_ of the ΔtssB-EP mutant was 1.0 × 10^8^ CFU/fish, representing a ~770-fold (2.7 log_10_) increase compared with the wild-type strain (1.3 × 10^5^ CFU/fish), indicating a marked change in phenotype, *i.e.*, attenuation of virulence. The cumulative mortality pattern was presented in **Figure 2**. To run a control, PBS was injected into healthy *L. rohita*.

### Serum IgM

**Figure 5:**
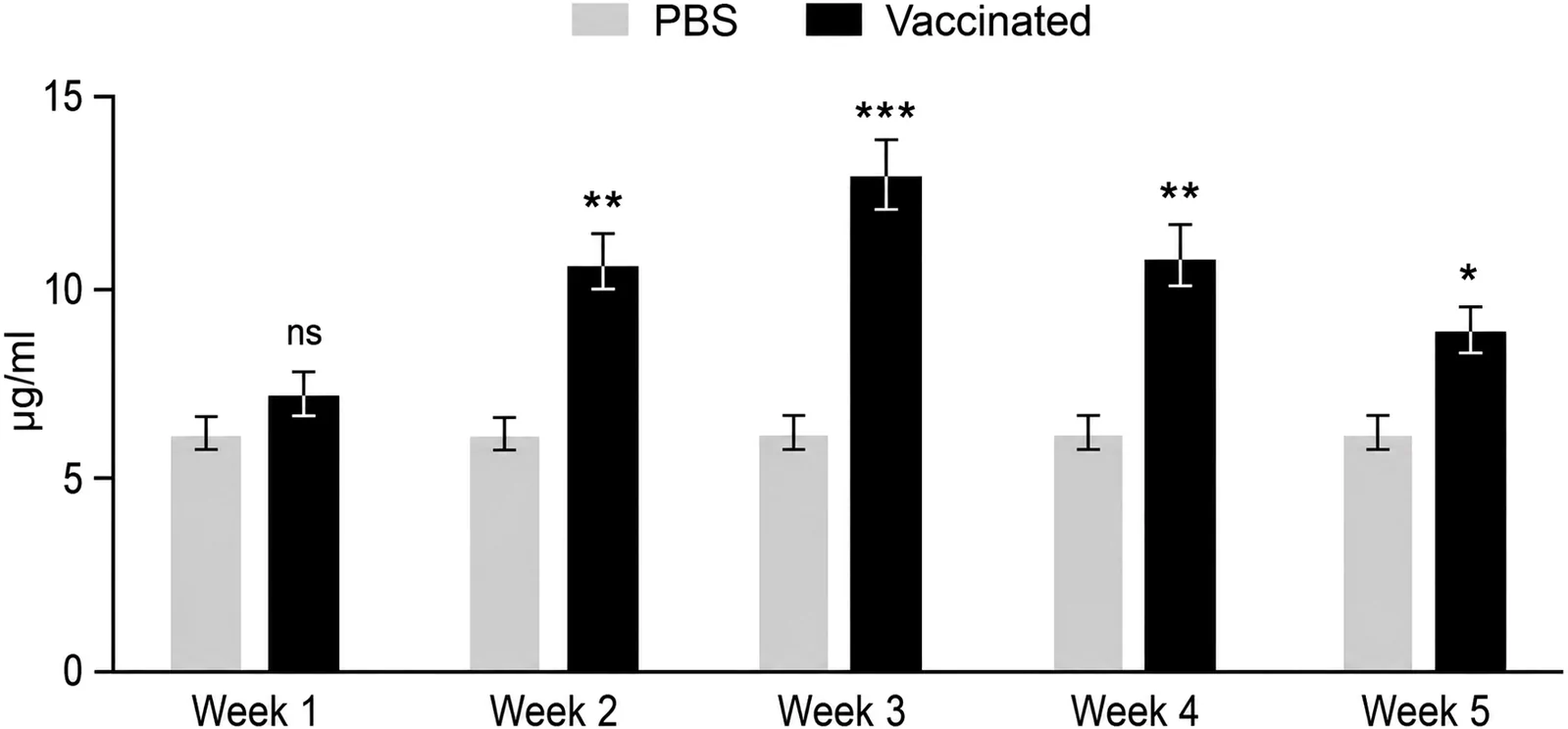
Total serum IgM levels in vaccinated *L. rohita* (n=6 at each collection time), estimated by ELISA over the immune induction period (weeks 1-5) using Cusabio Fish Immunoglobulin M (IgM) ELISA Kit. Mean (± standard error) is shown at each time point. *p<0.05, **p<0.01, ***p<0.001, ns=no significant difference, using two-way ANOVA, followed by Tukey’s multiple comparisons test. X-axis depicts time post-vaccination; y-axis, µg/ml of IgM.

The results showed that serum IgM levels were significantly different between the vaccinated group and the mean of the mock-vaccinated group serum IgM level (**Figure 5**). During the onset of the 3rd week or 21 days of post-vaccination (dpv), the antibody titer was at maximum, followed by the 4th week among the vaccinated group compared to mock-vaccinated or control (PBS). The assay measured total circulating IgM levels (26) in the serum of the tested fish.

### Relative percentage survival

**Figure 6:**
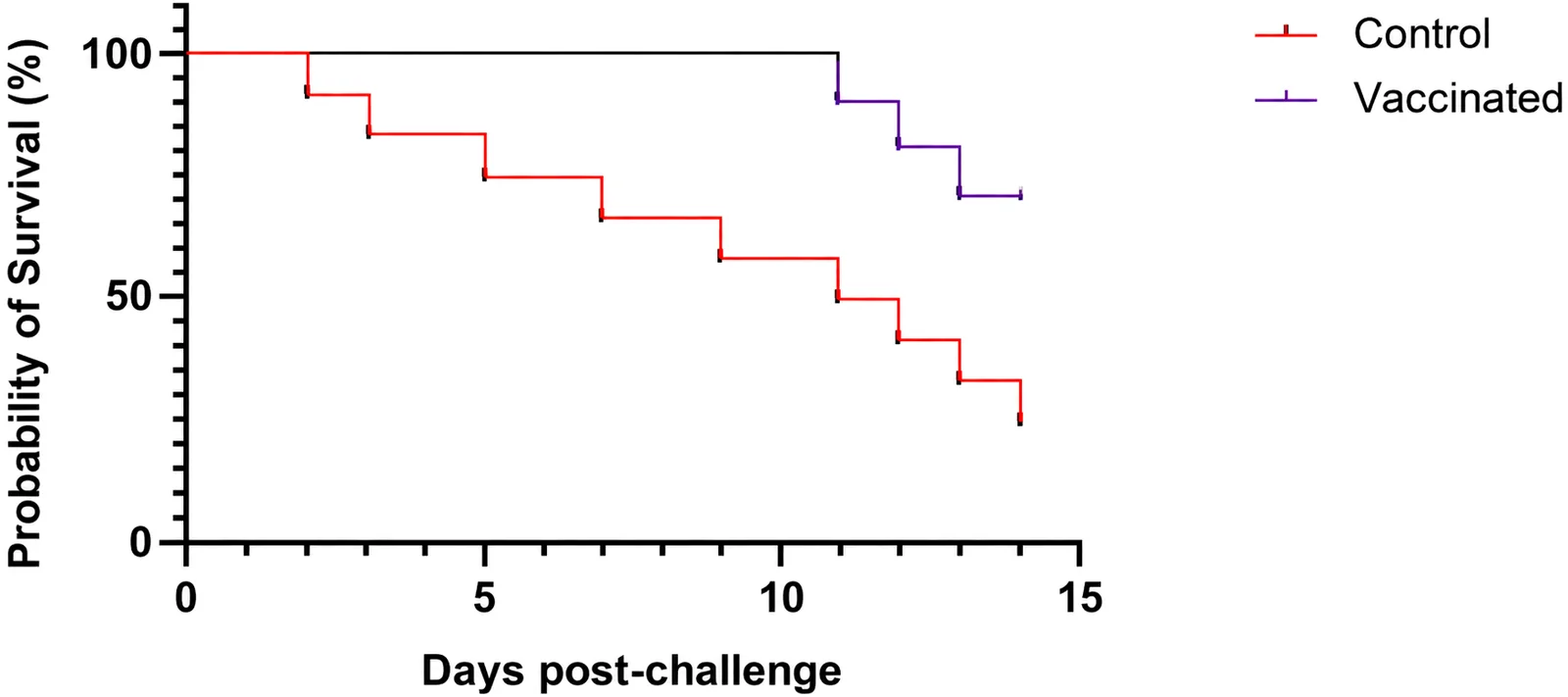
Kaplan-Meier survival plot of the control (Control) and vaccinated (Vaccinated) groups following ip challenge with wild-type *E. piscicida* at 1.0 × 10^8^ CFU/fish. Data represent the mean of 3 replicate tanks per group (n=20 fish per tank, 60 fish total per group). Days post-challenge are indicated (x-axis) and percent survival (y-axis).

No post-vaccination mortality was observed. A Kaplan-Meier survival analysis was conducted to assess mortality among *L. rohita* juveniles in vaccinated and unvaccinated groups following intraperitoneal challenge with the wild-type strain *E. piscicida* (WSFC2). Daily mortality was monitored, and *E. piscicida* was confirmed as the causative agent by re-isolating the bacterium from freshly deceased individuals. By 14 days post-challenge, the cumulative mortality in the vaccinated group was 30%, significantly lower than in the unvaccinated control group (P< 0.001; **Figure 6**), and RPS was 70.83%.

## Discussion

The principal finding of this study is that targeted deletion of *tssB*, which encodes a core structural component of the Type VI secretion system (T6SS), resulted in a clearly attenuated *E. piscicida* phenotype. The immunogenicity of Δ*tssB*-EP was retained and used to vaccinate *L. rohita* and conferred protection against mortality with 70.83% RPS. The mutant exhibited reduced biofilm formation, and the LD_50_ dose increased by 2.7 log_10_ relative to the wild-type strain. Taken together, *tssB* is an appropriate attenuation target, and Δ*tssB*-EP is a live attenuated vaccine candidate in *L. rohita.*

The rationale for selecting *tssB* is that TssB, also known as EvpB in *Edwardsiella*, is part of the contractile sheath of the T6SS apparatus and is required for functional secretion (15). T6SS is not a single toxin but a multiprotein delivery system involved in host-cell interaction, intracellular survival, immune modulation, and competition with other bacteria. Disruption of T6SS-related genes in *Edwardsiella* spp. has previously been associated with impaired virulence and reduced capacity to persist in host tissues (8, 10, 11). The present data are consistent with this concept: deletion of *tssB* did not simply abolish bacterial viability but altered virulence-associated phenotypes. This is key for a live vaccine candidate, and the challenge is to strike a balance between reducing virulence and maintaining the immunogenicity of the vaccine strain, thereby presenting a broad repertoire of antigens to the host immune system.

The virulence data showed a marked reduction in virulence for the Δ*tssB*-EP strain, indicating a relatively broad safety window for use as a vaccine. The graded dose-response pattern showed that mortality had a narrow temporal window, with deaths occurring predominantly during the first 3 days post-infection. While the ΔtssB-EP strain is markedly attenuated, there is residual virulence detectable at the highest inoculation doses. This is biologically plausible since *E. piscicida* possesses several virulence mechanisms, including intracellular survival strategies, adhesins, iron acquisition systems, stress-response pathways, and other secretion-associated factors (27). Loss of one central T6SS component is therefore expected to impair, but not necessarily eliminate, pathogenicity. In addition, the intraperitoneal route bypasses several natural barriers, including skin, gill, gut, and mucosal immune surfaces, and delivers bacteria directly into the body cavity and circulation. Mortality after high-dose intraperitoneal exposure may therefore overestimate the risk posed by the mutant delivered by other modalities, *i.e.*, immersion. Nevertheless, residual virulence is a relevant safety issue for a live attenuated vaccine and should be evaluated further by defining the maximum tolerated vaccine dose, persistence in host tissues, shedding, environmental survival, and safety across different fish sizes and physiological states. Alternative delivery routes need to be tested, which was not done in this study.

While safety is an important consideration, a limited degree of residual replication or persistence may contribute positively to vaccine efficacy. Live attenuated vaccines generally outperform killed or subunit preparations when they transiently mimic infection without causing overt disease, thereby allowing antigen presentation in a physiological context and stimulating both innate and adaptive immune pathways (28–30). The absence of mortality at the vaccine dose of 10^4^ CFU/fish, combined with subsequent protection against wild-type challenge, suggests that Δ*tssB*-EP lies within a potentially useful attenuation window, *e.g.*, sufficiently weakened to be tolerated at the administered dose, but still capable of stimulating protective immunity.

The likelihood of reversion to virulence needs to be considered. The Δ*tssB*-EP mutant was generated through an in-frame deletion rather than by chemical attenuation, serial passage, or a single point mutation. A defined deletion of approximately 506 bp in a structural T6SS gene is less likely to revert spontaneously than a point mutation, because restoration of the wild-type genotype would require reacquisition of the missing sequence or a homologous recombination event with an intact *tssB* locus. This makes true genetic reversion comparatively less likely. However, this does not remove all safety concerns. Compensatory mutations, regulatory changes, or the acquisition of genetic material from related bacteria could theoretically alter virulence-associated traits (31). Future work should therefore include serial *in vitro* and *in vivo* passages of the mutant, followed by PCR or whole-genome sequencing confirmation of the deletion, assessment of the absence of plasmid or antibiotic markers, and repeat virulence testing after passage. Such stability testing is essential before the strain can be considered for practical vaccine development.

The reduction in biofilm formation provides additional support for a virulence-attenuated phenotype. Biofilm formation may promote bacterial survival in host-associated and environmental niches, facilitate persistence under stress, and contribute to resistance against host defenses (32, 33). The lower OD_570_/OD_600_ ratio observed for Δ*tssB*-EP indicates that deletion of *tssB* affected biofilm-associated behavior and virulence. This phenotype may reflect disruption of T6SS-dependent interactions, altered surface behavior, or indirect changes in regulatory networks linked to colonization and persistence. However, the current biofilm assay is an *in vitro* endpoint and should be interpreted as supportive. Complementation of *tssB*, assays of intracellular survival in macrophage-like cells, and colonization studies in fish tissues would help distinguish whether reduced biofilm formation is directly linked to the attenuation mechanism or represents a parallel phenotype.

The immunological data indicate that vaccination with Δ*tssB*-EP induced a measurable humoral response. Serum IgM levels increased in vaccinated fish relative to PBS controls, with the strongest response occurring during the third week after vaccination and remaining elevated thereafter. However, the interpretation should be conservative since the assay measured total circulating IgM rather than antigen-specific anti-*E. piscicida* antibody. The data demonstrate only humoral activation but do not prove that the increased IgM is specific to *E. piscicida* antigens. Future studies should include an antigen-specific ELISA using whole-cell lysate, outer membrane proteins, or defined recombinant antigens, and ideally combine this with functional assays such as bacterial agglutination, opsonophagocytosis, or serum bactericidal activity.

The protection experiment provides important preliminary evidence of vaccine efficacy. Vaccinated fish showed substantially lower cumulative mortality than PBS-injected controls after challenge with virulent wild-type *E. piscicida*. This is a meaningful level of protection obtained for an early-stage live attenuated vaccine candidate against a high-dose intraperitoneal challenge. Notably, the challenge model tests systemic protection under stringent experimental conditions but does not replicate natural infection, in which exposure occurs through mucosal surfaces and colonization, invasion, and early innate defense mechanisms may strongly influence the outcome. The present result should therefore be read as a proof of concept for efficacy under controlled intraperitoneal challenge, while the effect under field conditions would require further scrutiny. Important factors to assess include, with particular focus, the duration of immunity and shedding into the environment; release into water systems and exposure of non-target organisms must be considered, not addressed in this study.

Overall, this study demonstrates that allelic-exchange deletion of *tssB* produces a genetically defined attenuation of *E. piscicida* with a strong reduction in virulence, reduced biofilm formation, detectable humoral immune activation, and significant protection against wild-type challenge. In addition to the obtained RPS, the demonstration that targeting a central T6SS structural component can shift the pathogen to a lower-virulence state while preserving vaccine potential is an important finding. The Δ*tssB*-EP mutant should therefore be viewed as a promising proof-of-concept candidate. Its further development will require complementation, genetic stability testing, safety assessment after passage, antigen-specific immune profiling, and efficacy testing under more natural routes of infection.

## Data Availability

The datasets presented in this study can be found in online repositories. The names of the repository/repositories and accession number(s) can be found in the article/**Supplementary Material**.
